# In Vitro Mechanical Stimulation to Reproduce the Pathological Hallmarks of Human Cardiac Fibrosis on a Beating Chip and Predict The Efficacy of Drugs and Advanced Therapies

**DOI:** 10.1002/adhm.202301481

**Published:** 2023-11-27

**Authors:** Roberta Visone, Camilla Paoletti, Alessandro Cordiale, Letizia Nicoletti, Carla Divieto, Marco Rasponi, Valeria Chiono, Paola Occhetta

**Affiliations:** ^1^ BiomimX Srl Milan 20157 Italy; ^2^ Department of Electronics Informatics and Bioengineering Politecnico di Milano Milan 20133 Italy; ^3^ Department of Mechanical and Aerospace Engineering Politecnico di Torino Turin 10129 Italy; ^4^ Centro 3R (Interuniversity Center for the Promotion of 3Rs Principles in Teaching and Research) Pisa 56122 Italy; ^5^ Istituto Nazionale di Ricerca Metrologica Division of Advanced Materials and Life Sciences Turin 10135 Italy

**Keywords:** cardiac fibrosis, direct reprogramming, efficacy screening, heart‐on‐chip, microRNAs

## Abstract

Cardiac fibrosis is one of the main causes of heart failure, significantly contributing to mortality. The discovery and development of effective therapies able to heal fibrotic pathological symptoms thus remain of paramount importance. Micro‐physiological systems (MPS) are recently introduced as promising platforms able to accelerate this finding. Here a 3D in vitro model of human cardiac fibrosis, named uScar, is developed by imposing a cyclic mechanical stimulation to human atrial cardiac fibroblasts (AHCFs) cultured in a 3D beating heart‐on‐chip and exploited to screen drugs and advanced therapeutics. The sole provision of a cyclic 10% uniaxial strain at 1 Hz to the microtissues is sufficient to trigger fibrotic traits, inducing a consistent fibroblast‐to‐myofibroblast transition and an enhanced expression and production of extracellular matrix (ECM) proteins. Standard of care anti‐fibrotic drugs (i.e., Pirfenidone and Tranilast) are confirmed to be efficient in preventing the onset of fibrotic traits in uScar. Conversely, the mechanical stimulation applied to the microtissues limit the ability of a miRNA therapy to directly reprogram fibroblasts into cardiomyocytes (CMs), despite its proved efficacy in 2D models. Such results demonstrate the importance of incorporating in vivo‐like stimulations to generate more representative 3D in vitro models able to predict the efficacy of therapies in patients.

## Graphical abstract

Developing effective treatment for cardiac fibrosis is essential. A 3D in vitro model is created by applying mechanical stimulation to human cardiac fibroblasts in a beating heart‐on‐chip. Mechanical stimulation induced fibrotic traits, which are prevented by anti‐fibrotic drugs, but highlights the limits of a miRNA‐therapy in reprogramming fibroblasts into CMs. In vivo‐like stimulation in 3D models is crucial to predict efficacy.

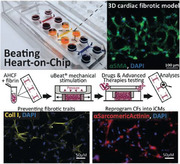

## 1 Introduction

Cardiac fibrosis remains one of the primary causes of end‐stage heart failure, which accounts for more than 10% of overall deaths worldwide.^[^ ^1^ ^]^ The identification of effective therapies able to contrast or to revert the symptoms of this pathology thus represents an urgent need in the drug discovery and development field.^[^ ^2^ ^]^ To achieve this goal, it is fundamental to get a deeper understanding of the disease process occurring in the myocardium during heart failure.^[^ ^3^ ^]^ Human adult heart cells possess a limited regenerative capacity; hence, after a cardiac insult, dead CMs are replaced by a permanent collagenous scar tissue which preserves the structural integrity of the heart. Although this reparative process is initially physiological, it often evolves into a pathological status, that causes thickening and stiffening of the heart walls, leading to chamber dilation, arrhythmias generation and ultimately conducting to heart failure.^[^ ^4^ ^]^ In this process, cardiac fibroblasts (CFs) play an essential role.^[^ ^5^, ^6^ ^]^ After CMs necrosis, the cardiac ECM is degraded and pro‐fibrotic signaling (among which the most important is the TGF‐β1 pathway^[^ ^7^ ^]^) is initiated, enhancing fibrotic remodeling. After an initial proliferative phase, resident CFs start to differentiate into their activated form, switching their phenotype into myofibroblasts. Myofibroblasts secrete a high quantity of collagen and other proteins, producing a fibrotic ECM with abnormal composition and quality that eventually impairs muscle function.^[^ ^8^ ^]^ In this scenario, 2D in vitro cell culture models have been extensively used in the last decades to study fibrotic remodeling and the role of both biochemical and biophysical pro‐fibrotic factors in the pathology.^[^ ^9^ ^]^ Specifically, biophysical cues have been investigated by tailoring the mechanical properties of cell culture supports or through the application of an active cyclic stretching to the cultures.^[^ ^8^, ^9^, ^10^, ^11^ ^]^ Nevertheless, 2D models often failed to recapitulate the native microenvironment and the biophysical stimuli that fundamentally contribute to the pathology, leading to imperfect or even misleading results on drug effects.^[^ ^10^, ^11^ ^]^ Similarly, animal models exploited to investigate the fibrotic remodeling process mainly fail in reproducing the human responses mostly because of interspecies differences.^[^ ^12^ ^]^ Therefore, despite some advances in dissecting the role of biochemical and biophysical stimulation in cardiac fibrosis,^[^ ^13^, ^14^, ^15^ ^]^ proper understanding of cardiac disease progression in humans is still limited, thus hampering the development of resolutive therapies.^[^ ^16^ ^]^ To overcome this issue, MPS have been proposed as advanced cell culture platforms able to combine 3D environment, chemical and physical stimulations, and human cells, with the aim to elucidate different aspects of cardiac fibrosis and serving as advanced in vitro models for drug screening.^[^ ^17^, ^18^ ^]^ A paramount example is the so called Biowire, that enabled to investigate the role of human CMs and CFs in the disease progression upon biochemical stimulation and that was exploited as pre‐clinical in vitro model for drug screening.^[^ ^18^, ^19^ ^]^ Other works focused on modulating the stiffness of human 3D cell culture environment (e.g., by tailoring the scaffold composition) to specifically assess the mechanical contribution to the onset of fibrotic traits and to propose treatments to mitigate them.^[^ ^20^, ^21^, ^22^ ^]^ Mechano‐transduction in cardiac fibrosis has emerged indeed as a fundamental aspect to be considered since mechanical function of CFs actively regulates heart growth, homeostasis, repair and disease.^[^ ^23^ ^]^ Besides TGF‐β, different pathways have been proposed for the cardiac fibrosis process mediated by mechano‐transduction: the transcriptional regulation of the Wnt/β‐catenin and Hippo pathways are involved in mechanical forces exerted at cell–cell adhesion level,^[^ ^24^ ^]^ mechanosensitive stimulation activates the RhoA/ROCK pathway^[^ ^25^ ^]^ and mechanical strain or stress on cardiac tissue, like hypertension, can activate the β‐catenin/Wnt signaling pathway.^[^ ^26^ ^]^

In vitro mechanical stimulation has been also frequently applied to drive CMs maturation. Indeed, CMs in native cardiac tissue are subjected to several kinds of stimuli,^[^ ^27^ ^]^ among which the mechanical one starts from early embryogenesis. Uniaxial cyclic strain has been reported to guide CM alignment, gene and protein expression toward a mature phenotype and cell interconnection through gap junction expression.^[^ ^28^ ^]^ Hence, cyclic mechanical stimulation deserves investigation not only for its involvement in the fibrotic process, but also as an additional physical cue to enhance in vitro direct reprogramming efficiency of cardiac fibroblasts into mature induced cardiomyocytes (iCM) as a possible therapeutic strategy to counteract the pathological states. Recent studies indeed have highlighted the role of a physiological‐like culture microenvironment (i.e., a cellularized 3D matrix with ECM‐mimetic composition and stiffness) in regulating cell fate, enhancing direct reprogramming of fibroblasts into iCMs.^[^ ^29^, ^30^ ^]^ However, so far the effect of mechanical stimulation on direct reprogramming of fibroblasts into iCMs has been only investigated by Sia et al.^[^ ^31^ ^]^ who demonstrated that cyclic mechanical stimulation of fibroblast cultures negatively influences their direct reprogramming into iCMs compared to static cultures. The authors hypothesized that mechanical stimulation could be useful only after the phenotypic switch of fibroblasts into iCMs, with the aim to support iCM maturation, rather than in early phases post‐transfection. Despite this initial study, the role of mechanical stimulation in direct reprogramming of fibroblasts into iCMs has not been further investigated.

To elucidate the role of mechanical stimulation both in cardiac fibrosis onset and prevention, and to investigate its effect on direct reprogramming of fibroblasts into iCMs, herein we present a 3D in vitro model of human cardiac fibrosis, named uScar, developed by imposing a cyclic mechanical stretching to AHCFs, cultured in uBeat Stretch Platform. uBeat Stretch Platform has been designed to provide a physiological uniaxial strain of 10% to 3D microtissues, triggering in vitro the main steps of cardiac fibrosis, such as fibroblast‐to‐myofibroblast transition and ECM deposition. The model was first validated as suitable to perform drug screening tests, confirming the efficacy of well‐known anti‐fibrotic compounds in preventing the onset of pathological traits. Mechanically induced uScar was then applied to study the efficacy of a new strategy focusing on contrasting cardiac fibrosis by directly reprogramming AHCFs into iCMs. To this aim, a previously reported combination of four microRNAs,^[^ ^30^, ^32^ ^]^ called miRcombo (miR‐1, miR‐133, miR‐208, and miR‐499) was delivered to AHCFs by recently developed lipoplexes.^[^ ^33^ ^]^ Two complementary experiments were performed with the aim to predict in vivo direct reprogramming outputs, elucidating: i) the capability of the therapy to prevent fibrotic traits in a mechanically active environment (i.e., cyclic mechanical stimulation applied to miRcombo transfected cell); ii) the potential to use the therapy to revert fibrotic traits (i.e., reprogramming performed in situ in a mechanically induced 3D cardiac fibrotic microenvironment). In both cases, the efficacy of miRcombo therapy was hampered by the mechanical stimulation, but not by the static 3D environment, suggesting the importance of screening therapies in relevant tissue models, recapitulating both 3D architecture and mechanical microenvironment.

## 2 Results

### 2.1 Fibrotic Cardiac Model Generation within uBeat Stretch Platform

**Figure 1:**
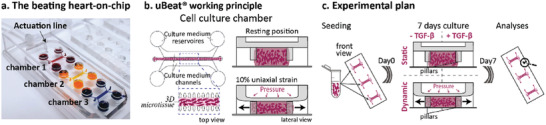
Fibrotic human cardiac model development: a) uBeat Stretch Platform encompassing 3 biologically independent cell culture chambers (red, yellow, blue) with a common actuation line; b) Working principle of the uBeat technology: the cell culture chamber houses two rows of hanging shield‐shaped pillars that i) confine a 3D microtissue (i.e., cell‐laden hydrogel) flanked by two lateral medium channels (top view) and ii) enable the application of 10% uniaxial strain to the cultured microtissues (lateral view); c) Experimental plan followed to achieve the generation of the models.

The human 3D cardiac models were developed within uBeat Stretch Platform (BiomimX Srl),^[^ ^34^, ^35^ ^]^ which encompasses three biologically independent cell culture chambers (**Figure** **1**a, blue, yellow and red) sharing a unique actuation compartment for the provision of the mechanical stimulation. The platform is constituted by 3 different layers: a top actuation chamber, an intermediate cell culture chamber and a bottom coverslip (Figure S1a, Supporting Information). Each cell culture chamber hosts a 3D microtissue (i.e., 300 µm wide, 150 µm tall and 8 mm long) in its central channel, where the fibroblast‐laden fibrin gel is confined by two rows of overhanging posts, allowing the supply of the cell culture medium from the flanking lateral channels (Figure 1b). The uniaxial strain stimulation is ensured by the uBeat technology:^[^ ^36^ ^]^ the actuation compartment is pressurized so to deform the top part of the cell culture chambers, pushing the hanging posts toward the coverslide (Figure 1b). Specifically, a 0.4 bar pressure was sufficient to impart the cardiac microtissue an uniaxial strain of about 7–12% in the different cell culture chambers of the device, with a mean value of 10% (Figure 1b,c, Supporting Information). To drive the onset of fibrotic traits, human atrial cardiac fibroblasts were laden into fibrin gel and cultured within uBeat Stretch Platform for 7 days while subjected to 3 different stimulations (Figure 1c): 1) an hyper‐physiological biochemical pro‐fibrotic stimulation supplementing the media with 5 ng mL^−1^ of TGF‐β1 (namely “Static + TGF‐β1”), b) a physiological mechanical stimulation providing a cyclic 10% stretching at 1 Hz (namely “Dynamic”), and 3) a combination of mechanical and biochemical stimulation (namely “Dynamic + TGF‐β1”). Static culture was used as control. Cell viability was preserved in both static and dynamic culture conditions (Figure S2, Supporting Information).

### 2.2 uScar Model: Fibroblast‐to‐Myofibroblast Transition and Alteration of the Gene Profile

**Figure 2:**
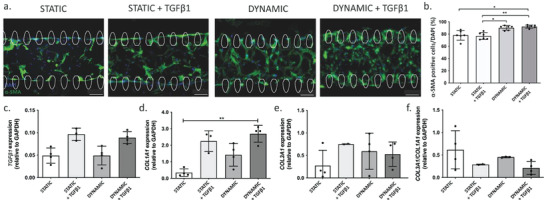
Fibroblast to myofibroblast transition in the human cardiac model subjected to chemical (TGF‐β1) and/or mechanical stimulations: a) Representative immunofluorescence images of myofibroblasts, identified by a‐SMA staining (green); b) Quantification of α‐SMA positive cells as percentage of the total number of cells in the microtissue. Data are represented as Mean ± SD (*n* = 6). Cardiac fibrosis markers in the human models among the four different experimental groups: mRNA level expression normalized to GAPDH of c) Tgfβ1, d) Collagen type‐I (Col1a1), e) Collagen type‐III (Col3a1), and f) their ratio (Col3a1/Col1a1) (data calculated with 2‐ΔCt method). Scalebar 100 µm. Data are represented as mean ± SD (*n* >= 3). Kruskal‐Wallis analysis of variance and Dunn's post‐test (* = *p* < 0.05, ** = *p* < 0.01).

The main stages of the cardiac fibrosis onset and progression involve fibrotic pathways activation, which in turn leads to a fibroblast‐to‐myofibroblast transition and triggers an excessive ECM deposition.^[^ ^8^ ^]^ The fibroblast‐to‐myofibroblast transition was assessed after 7 days in culture within uBeat Stretch Platform. Immunofluorescence images of alpha Smooth Muscle Actin (α‐SMA) stained myofibroblasts (**Figure** **2**a) qualitatively showed that few myofibroblasts were present in static conditions, with a slight increase when TGF‐β1 was added. The cyclic mechanical stimulation instead significantly contributed to fibroblast phenotype transition into myofibroblasts, as evidenced by the representative images. Quantification of the ratio between α‐SMA positive cells and cell nuclei confirmed the significant effect of cyclic mechanical stimulation in triggering fibroblast‐to‐myofibroblast transition in both “Dynamic” (90.2 ± 3.7%, *p* = 0.046) and “Dynamic + TGF‐β1” conditions (92.2 ± 2.5%, *p* = 0.0068), with respect to “Static + TGF‐β1” (76.8 ± 5.7%) (Figure 2b). Noteworthy, the supplementation of TGF‐β1 did not induce changes in the percentage of α‐SMA positive cells in both static (76.8 ± 5.7% versus 78.0 ± 7.9% with no TGF‐β1) and dynamic (92.2 ± 2.5% versus 90.2 ± 3.7% with no TGF‐β1) conditions. Hence, the combined stimulation activated the cells at a similar level with respect to the mechanical stimulation alone, statistically increasing the α‐SMA positive cells with respect to the static condition (*p* = 0.014).

Real time q‐PCR revealed that *TGFB1* mRNA expression (Figure 2c) increased when the chemical stimulation was provided either alone (twofold increased respect to static) or in combination with dynamic conditions (1.8‐fold increased respect to static and to dynamic groups). Conversely, no changes in *TGFB1* mRNA expression were evidenced in microtissues mechanically stimulated with respect to the controls. Despite the trends, no significant differences were observed between the experimental groups. The supplementation of TGF‐β1 also showed an enhanced mRNA expression of both Collagen type I (*COL1A1*) and Collagen type III (*COL3A1*) fibrosis markers, resulting in 6.5‐ and 2.7‐fold increases respectively, compared to the static group (Figure 2d,e). Despite at lower level (four and twofold increases), also the mechanical stimulation enhanced the expression of both collagens‐related genes compared to static microtissues. Moreover, the combined stimulation statistically increased the expression level of *COL1A1* (7.7‐ fold increase, *p* = 0.0094) and enhanced *COL3A1* expression (twofold increase) respect to the controls. However, when compared to the individually applied stimuli, the combined stimulation only slightly contributed to enhance *COL1A1* (1.2‐ and 1.9‐fold increases) and even mildly reduced *COL3A1* expressions (0.7‐ and 0.9‐fold increase) compared to microtissues administered with TGF‐β1 or subjected to cyclic uniaxial strain, respectively. The ratio between *COL3A1* and *COL1A1*, whose unbalance is a typical hallmark of cardiac fibrosis, resulted increased when the mechanical stimulation was applied alone respect to static or dynamic cultures supplemented with TGF‐β1. However, all the levels remained lower if compared to the controls (Figure 2f).

### 2.3 uScar Model: Enhanced ECM Deposition

**Figure 3:**
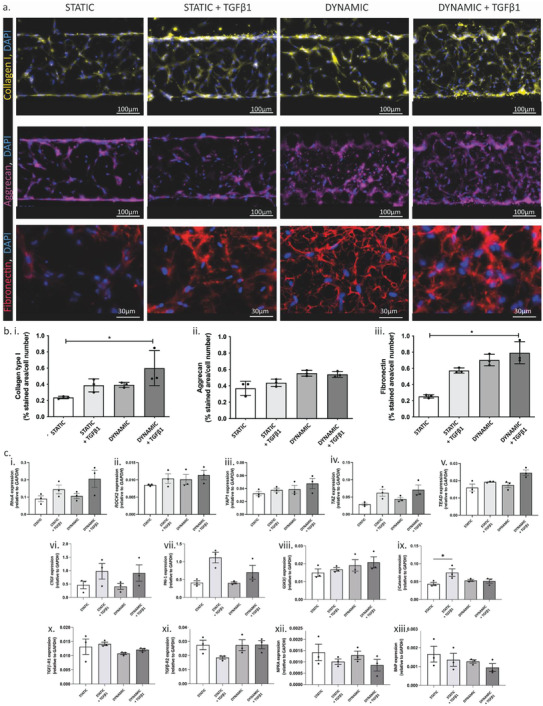
ECM components deposition in the human cardiac model after 7 days of culture in the four different experimental groups: a) Representative images of immunofluorescent staining for Collagen I (yellow), Aggrecan (Purple) and Fibronectin (red); b) Quantification of Collagen I, Aggrecan, and Fibronectin production, calculated as the ratio between the percentage of stained area and the total number of cell nuclei. Scalebar 100 µm for Collagen I and Aggrecan and 30 µm for Fibronectin. Data are represented as mean ± SD (*n* >= 3). Kruskal‐Wallis analysis of variance and Dunn's post‐test (* = *p* < 0.05). c) Droplet digital PCR analysis of RhoA/ROCK2 and Hippo pathway‐associated genes in the human models among the four different experimental groups: mRNA level expression normalized to GAPDH of i) RhoA, ii) ROCK2, iii) YAP1, iv) TAZ, v) TEAD, vi) CTGF, vii) PAI‐1, viii) GSK3b, ix) b‐catenin, x) TGFb‐R1, xi) TGFb‐R2, xii) NPRA and xiii) BNP. Data are represented as mean ± SD (*n* >= 3). Kruskal‐Wallis analysis of variance and Dunn's post‐test (* = *p* < 0.05).

ECM deposition was evaluated after 7 days of culture in different conditions through immunofluorescence staining. In **Figure** **3**a, representative images of Collagen type I (first row, yellow) in combination with DAPI nuclei counterstaining (blue), showed that in static condition fibroblasts produced low Collagen type I amount, while an exogenous addition of TGF‐β1 or the application of the cyclic uniaxial strain (alone or combined with the chemical stimulation) enhanced protein production, especially in the region nearby the pillars. The qualitative observations were confirmed by quantification of the Collagen type I deposition, estimated as a ratio between the stained area (%) and the number of cell nuclei (Figure 3b,i). All the applied stimulations indeed enhanced Collagen type I production, with a statistically significant improvement (*p* = 0.013) observed when microtissues were mechanically stimulated and supplemented with TGF‐β1, respect to the controls. In static conditions, Aggrecan (Figure 3a, second row, purple) was present mainly around the pillars and near the cell nuclei, indicating a prevalence of intra‐cellular protein production. The addition

of TGF‐β1 or the dynamic stimulation triggered a more homogeneous production of Aggrecan, which appeared distributed throughout the entire microtissue. Aggrecan quantification showed increased proteoglycan level on the microtissue which had been cyclically stretched both with or without TGF‐β1 supplementation (Figure 3b,ii). Similarly, Fibronectin deposition (Figure 3a, third row, red) was enhanced both by TGF‐β1 administration or by mechanical stimulation (alone or combined with the biochemical factor), with a homogenous matrix deposition throughout the whole construct. In all the conditions, a huge deposition of the protein was also detected around the pillars (Figure S3, Supporting Information). Quantitative data showed an enhanced production of Fibronectin in all the stimulated groups as compared to the static controls, especially in microtissues mechanically stimulated in the presence of TGF‐β1 factors, reaching statistically significant higher level (*p* = 0.028) (Figure 3b.iii).

### 2.4 Pathways Involved in the uScar Model

To identify critical genes involved in inducing cell transition into myofibroblast phenotype and increased ECM deposition through mechanical strain, we investigated pathways known to be activated in response to mechanical cues, such as RhoA/ROCK and Hippo pathways. As expected, we found increased RhoA (Figure 3c,i) and ROCK2 (Figure 3c,ii) expression, although not significant, in microtissues treated with TGF‐β1 compared to static condition alone. Indeed, these genes are known to be activated by TGF‐β pathway.^[^ ^37^ ^]^ The combination of TGF‐β1 administration and cyclic uniaxial strain was found to further increase RhoA expression in the microtissues, suggesting that the two stimuli have a synergistic effect on RhoA activation. Moreover, ROCK2 expression was enhanced in microtissues cultured in both dynamic conditions (with and without TGF‐β1 administration) compared to static one. Regarding the Hippo pathway, we observed not significant, but increased expression of YAP, TAZ and TEAD in microtissues cultured in dynamic conditions or treated with TGF‐β1 (Figure 3c,iii–v). The combination of both stimuli was able to further increase the expression of Hippo‐associated genes compared to the two stimuli alone and static culture condition. CTGF and PAI‐1 gene expression was enhanced by the TGF‐b1 administration (Figure 3c,vi,vii). Uniaxial strain, either by itself or in combination with TGF‐β1, increased the expression of GSK3 compared to microtissues cultured under static conditions or treated with TGF‐β1 alone (Figure 3c,viii), suggesting an involvement of WNT∖β‐catenin pathway.^[^ ^38^ ^]^ However, it did not impact the expression of β‐catenin, which exhibited a statistically significant upregulation only when exposed to TGF‐β1 treatment (Figure 3c,ix). Concerning the TGF‐β pathway, in our model the expression levels of TGF‐β receptors, specifically TGFβ‐R1 and TGFβ‐R2, remained unchanged in both the dynamic and dynamic + TGFβ1‐stimulated conditions compared to the control group (Figure 3c,x,xi). Conversely, the expression of BNP decreased in both the dynamic and dynamic + TGF‐β1 conditions, and the expression of NPRA diminished when both stimulations were combined, confirming the previously reported involvement of BNP and NPRA in modulating myocardial fibrosis^[^ ^39^ ^] (^Figure 3c,xii,xiii). Overall, the presented results demonstrated that the mechanical stimulation alone is sufficient to trigger fibrotic traits in the model, enhancing the number of myofibroblasts and increasing the matrix deposition at similar or superior levels respect to the canonical TGF‐β1 administration, which may correlate with RhoA/ROCK and Hippo signaling pathway activation.

### 2.5 Anti‐Fibrotic Drugs Prevent Fibrotic Trait Onset in the uScar Model

**Figure 4:**
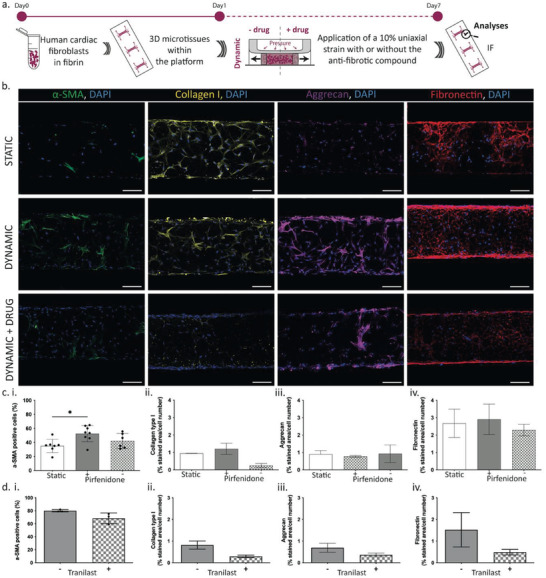
Effect of drugs in preventing the fibrotic traits elicited by the mechanical stimulation in the uScar human model: a) Schematic representation of the experimental plan; b) Representative immunofluorescence images of activated fibroblasts (α‐SMA‐green) and ECM components, such as Collagen I (yellow), Aggrecan (purple) and Fibronectin (Red) and c) relative quantification when Pirfenidone is administered (horizontal dotted line refers to the static controls); d) phenotype switch and ECM deposition quantification when Tranilast is administered. The ECM component quantification was derived as the ratio between the percentage of stained area and the total number of cell nuclei. Scale bar:100 µm. Data are represented as mean ± SD. Kruskal‐Wallis analysis of variance with Dunn's post‐test for all the data, except for α‐SMA with Pirfenidone, analyzed by Ordinary one‐way Anova multiple comparison test after verifying the normality of the dataset.

To qualify uScar as preclinical model for screening the efficacy of anti‐fibrotic compounds, the role of two drugs (i.e., Tranilast and Pirfenidone) with a known effect in preventing the onset of pathological features was evaluated. Specifically, the drugs were dissolved in culture medium and administered for the entire culture period while the microtissues were subjected to mechanical stimulation (**Figure** **4**a). After 7 days of culture, myofibroblasts proportion and ECM deposition were evaluated through immunofluorescence staining. The administration of 1 mM of Pirfenidone, for which representative images are reported in Figure 4b, resulted able to limit the phenotype switch of CFs triggered by mechanical cues (Figure 4b, green first column). The number of α‐SMA positive cells appeared statistically lower with respect to dynamic condition (*p* = 0.015) and more comparable with the static control (Figure 4c,i). Also ECM deposition was hindered by Pirfenidone, which drastically reduced Collagen type I production, as clearly highlighted from immunofluorescence analysis (Figure 4b, yellow second column). Specifically, Pirfenidone reduced Collagen type I production with respect to both dynamic (5 time less) and static (4 time less) controls (Figure 4c,ii). Moreover, while the drug only mildly affected Aggrecan production (Figure 4b, purple third column), it was efficient in decreasing mechanically‐induced Fibronectin deposition (Figure 4b, red fourth column). In more detail, the drug decreased both the levels of Aggrecan (i.e., 14% and 17%) (Figure 4c,iii) and of Fibronectin (i.e., 14% and 21%) respect to microtissues cultured in dynamic and static conditions, respectively. Similarly, the administration of 50 µm of Tranilast resulted effective to lower the phenotype transition of fibroblasts into myofibroblasts and to reduce the ECM deposition, as shown by immunofluorescence staining of myofibroblast marker α‐SMA and ECM protein content such as Collagen type I, Aggrecan and Fibronectin reported in the Figure S4, Supporting Information. Particularly, Tranilast reduced of about 30% the number of activated fibroblasts (Figure 4d,i) and limited mechanically‐induced ECM deposition. Collagen type I production indeed was diminished of about 60% (Figure 4d,ii) and Aggrecan and Fibronectin were hugely reduced of about 66% and 50%, respectively, as compared to the dynamic condition without the drug (Figure 4d,iii,iv).

### 2.6 Mechanical Stimulation Impairs the Efficacy of Nanotherapeutics in Preventing Fibrotic Traits Onset, Hampering the Direct Fibroblast Reprogramming into iCMs

**Figure 5:**
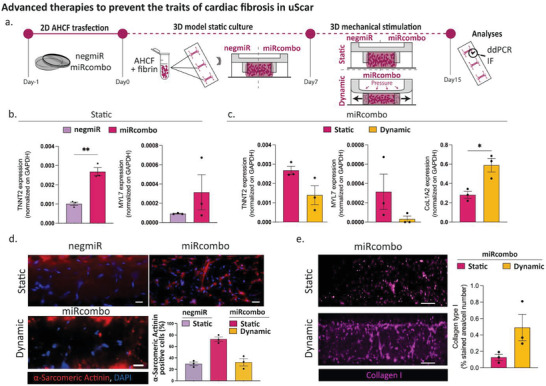
Study of miRcombo‐mediated direct reprogramming of AHCFs into iCMs with/without cardiac tissue‐like cyclic mechanical stimulation in the uScar human model: a) Schematic representation of the experimental plan; b) mRNA level expression of cardiomyocyte typical markers (TNNT2, MYL7) in static samples obtained from AHCFs transfected with negmiR or miRcombo after 15 days of culture; c) mRNA level expression of TNNT2, MYL7 and fibrotic marker COL1A2 in static or dynamic samples obtained from AHCFs transfected with miRcombo after 15 days of culture; Representative immunofluorescence images of d) a‐sarcomeric actinin cardiomyocytes‐related protein (red) with DAPI counterstaining (blue) and of e) Collagen I fibrosis marker (purple) with relative quantification in static and dynamic samples obtained from AHCFs transfected with miRcombo after 15 days of culture. Scalebar 50 µm for a‐Sarcomeric Actinin and 100 µm for Collagen I. Data are presented as mean ± SEM. Statistical differences between the groups were determined with two‐sided *t*‐tests.

Transient transfection with miRcombo, a combination of a four microRNAs (miR‐1, 133, 208, and 499) was previously found able to trigger the direct reprogramming of mouse and human fibroblasts into cardiomyocyte‐like cells, both in 2D and 3D macroscale culture conditions, using commercial DharmaFECT1 transfection agent.^[^ ^30^, ^32^ ^]^ In a more recent work, direct cell reprogramming efficiency was found to increase by delivering miRcombo to AHCFs using [2‐(2,3‐didodecyloxypropyl)‐hydroxyethyl] ammonium bromide (DE) and L‐alpha‐dioleoylphosphatidylethanolamine (DOPE) liposomes to form DE‐DOPE/miRcombo lipoplexes^[^ ^33^ ^]^that were thus exploited in this study. In a first experiment, cells were transfected with miRcombo or negmiR for 24 h in 2D conditions and then cultured in 3D fibrin hydrogel in the uBeat Stretch Platform for 1 week in static condition, followed by additional 7 days culture in static or dynamic (i.e., cyclic uniaxial strain of 10%, 1 Hz) condition (**Figure** **5**a).

As expected from previous findings,^[^ ^32^ ^]^ AHCFs transfected with miRcombo and cultured in 3D for 15 days in static condition showed a statistically enhanced expression of *TNNT2* (almost twofold, *p* = 0.0071) and an improved level of *MYL7* genes, which are typical markers of cardiomyocytes, respect to negmiR‐transfected cells (Figure 5b). Conversely, miRcombo‐transfected AHCFs showed a significantly lower conversion into cardiomyocyte‐like cells when cultured in dynamic conditions (Figure 5c). Indeed, *TNNT2* gene resulted downregulated and the expression level of *MYL7* gene was also reduced in dynamic culture condition with respect to the static controls. On the other hand, *COL1A2* gene, a marker of cardiac fibrosis was significantly upregulated (twofold, *p =* 0.005) in microtissues subjected to cyclic mechanical strain with respect to samples cultured in static condition. Results were confirmed by protein expression analysis (Figure 5d,e). Immunofluorescence of Alpha‐Sarcomeric Actinin, a constituent protein of cardiomyocyte sarcomeres, showed a spread expression in miRcombo‐transfected cells compared to negmiR ones cultured in static conditions, respectively, 70% versus 30% of α‐Sarcomeric Actinin positive cells (Figure 5d). Conversely, mechanical stimulation induced a sporadic production of Alpha‐Sarcomeric Actinin (30% of α‐sarcomeric actinin positive cells), merely near the pillars, while negmiR transfection followed by culture in static condition did not trigger Alpha‐Sarcomeric Actinin expression (data not showed) (Figure 5d). Similarly, Collagen type I expression, marker of fibrosis, was around fourfold higher in dynamically cultured samples with respect to static culture condition, confirming that mechanical stimulation acts as a pro‐fibrotic factor and reduces miRcombo efficacy (Figure 5e).

### 2.7 Nanotherapeutics Partially Reverse Fibrotic Traits Induced in the uScar Model, Despite the Low Reprogramming Efficiency

**Figure 6:**
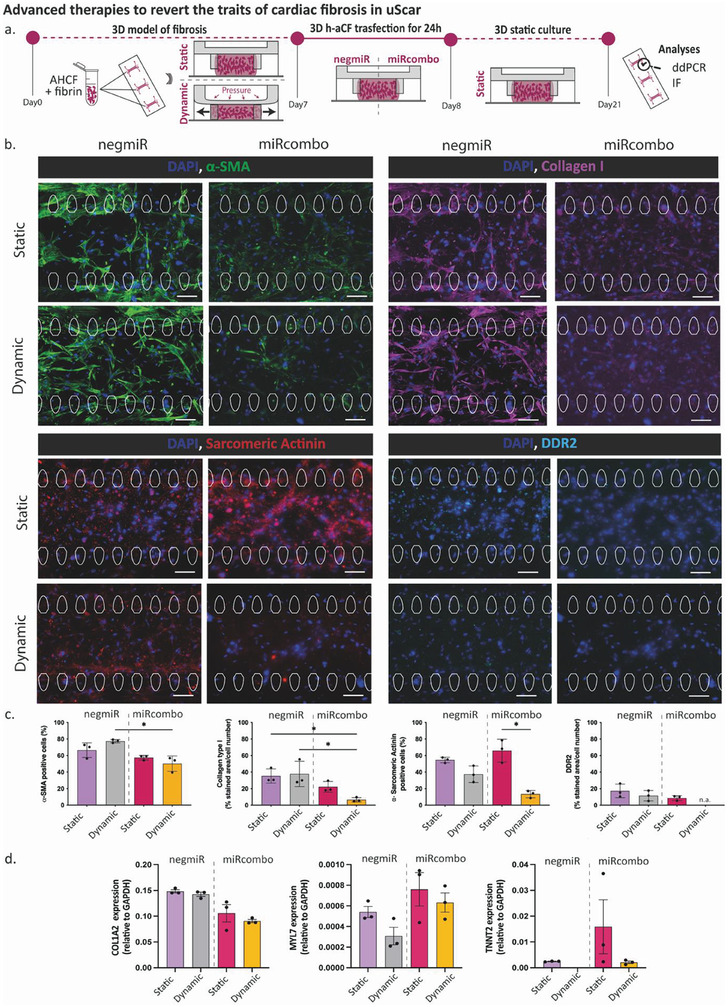
Effect of the advanced gene therapy in reverting the fibrotic traits elicited by the mechanical stimulation in the uScar human model: a) Schematic representation of the experimental plan; b) Representative immunofluorescence images of α‐SMA (green), collagen I (purple), α‐Sarcomeric Actinin (red), DDR2 (light blue) and DAPI counterstaining (blue) in the four experimental groups and c) relative quantifications. Scalebar 100 µm. Data are represented as mean ± SD. Kruskall‐Wallis analyses of variance with Dunn's post‐test was used (* = *p* < 0.05, ** = *p* < 0.01, *** = *p* < 0.001).

To assess the efficacy of the therapy to revert fibrotic pathological traits, miRcombo transient cell transfection through DE‐DOPE lipoplexes was directly performed in the preformed uScar model. Specifically, the microtissues were mechanically stimulated or statically cultured for 1 week, then transfected with miRcombo or negmiR for 24 h directly on chip and finally cultured for an additional 2 weeks in static condition (**Figure** **6**a). The treatment with miRcombo successfully reduced the fibrotic traits of microtissues, previously induced by mechanical stimulation on the uBeat Stretch Platform. However, miRcombo did not effectively induce a transition to a CM‐like phenotype of the mechanically stimulated cells at 14 days post transfection (Figure 6b,c). Specifically, the administration of miRcombo to mechanically stimulated microtissues resulted in a statistically significant decrease in the number of α‐SMA positive cells (*p* = 0.04) and deposition of Collagen I (*p* = 0.02) compared to cells that were mechanically stimulated and treated with negmiR. The trend, despite not at statistically significant level, was confirmed by gene expression analyses of COL1A2 performed 14 days post transfection (21d in culture) (Figure 6d). As expected, *MYL7* gene expression resulted slightly more expressed in the miRcombo conditions compared to negmiR (1.5‐fold for static and twofold for dynamic microtissues). However, the mechanical stimulation appeared to slightly reduce the *MYL7* gene expression compared to static controls both in negmiR (1.7‐fold decreased) and miRcombo (i.e., 1.2‐fold decreased) treated microtissues. Nevertheless, the measurement of α‐Sarcomeric Actinin showed that under dynamic conditions, the effectiveness of miRcombo in inducing iCMs was significantly reduced (fivefold reduction, *p* = 0.027) compared to microtissues cultured in a static environment. Despite not at significant levels, miRcombo appeared to mitigate fibrotic traits also in static conditions (i.e., lower α‐SMA positive cells and Collagen I deposition) and to induce a higher iCM transition (i.e., higher α‐Sarcomeric Actinin positive cells respect to negmiR). DDR2, a fibroblasts/myofibroblasts’ collagen‐receptor marker, was mildly present in all the conditions (except for miRcombo in dynamic conditions), matching the trend of Collagen I. DdPCR results performed after 7‐days post transfection revealed that mechanically active microtissues showed a downregulated TNNT2 gene expression both when treated with miRcombo or with negmiR respect to statically cultured and transfected models (2.8‐ and 2.4‐fold decrease respectively, *p =* 0.014 and *p =* 0.045) (Figure S5a, Supporting Information). The trend was confirmed in gene expression analyses 14‐days post transfection (Figure 6d). Notably, tissue models developed in static culture condition showed a slight enhanced TNNT2 gene expression after miRcombo administration 7‐days post transfection with respect to negmiR controls (FigureS5a, Supporting Information), that hugely increased after 14‐days post transfection (Figure 6d). After 7 days post transfection, cardiac Troponin T was mildly present at similar levels in all the treated microtissues, including the ones cultured in static control (Figure S5b, Supporting Information).

## 3 Discussion

In this work we described the development of a miniaturized human cardiac fibrotic model, uScar, that was generated by applying mechanical stimulation to human cardiac fibroblasts cultured in 3D. The sole provision of a cyclic 10% uniaxial strain at 1 Hz induced fibrotic traits in the microtissues. The model resulted suitable as a tool to investigate the efficacy of drugs and a miRNA therapy in preventing or reverting the fibroblast‐mediated scar‐like tissue formation. Compared to previously developed platforms,^[^ ^35^ ^]^ the current assembly of the actuation and cell culture layers, allowed to develop microtissues in direct contact with the bottom coverslip, ensuring a better optical inspection of the samples even at high magnifications. Nevertheless, the uBeat technology working principle was maintained and the expected level of physiological deformations (i.e., 10% strain^[^ ^35^ ^]^) was achieved by operating the actuation compartment with similar pressure level. AHCFs embedded in fibrin hydrogel subjected to biochemical (i.e., TGFβ1) and/or mechanical stimulation were capable to recapitulate the initial stages of cardiac fibrosis, involving the fibroblast phenotype switch into myofibroblasts and enhanced ECM deposition.^[^ ^40^ ^]^ Specifically in our model, after 7 days of culture, cyclic mechanical strain resulted as the principal trigger for the induction of fibroblast‐to‐myofibroblast transition (Figure 2a,b). The addition of TGF‐β1 also triggered fibroblast to myofibroblast transition but at a lower extent, unless combined with mechanical stimulation, despite such cytokine is known to be involved in cardiac fibrosis and broadly used as a gold standard to induce fibrosis in in vitro models.^[^ ^41^ ^]^ This suggested that in our set‐up, the cyclic mechanical stimulation alone played a key‐role in fibroblasts activation. As expected, exogenous TGF‐β1 administration enhanced the cytokine gene expression while it was not increased by the dynamic stimulation alone (Figure 2c). Such results demonstrated that mechanical stimulation‐mediated transition of fibroblasts into myofibroblasts is partially independent from the TGF‐β pathway. Therefore, other mechanosensing‐dependent or inflammation‐related pathways were investigated to understand pro‐fibrotic cell activation.^[^ ^23^ ^]^ TGF‐β1 enhanced *COL1A1* expression, especially when combined with mechanical stimulation, while COL3A1 expression remained highly variable within each group and comparable among different conditions (Figure 2d,e). The decreased ratio between *COL3A1* and *COL1A1* expression suggested that, in all conditions, there was a superior accumulation of new Collagen Type I respect to Collagen Type III (Figure 2f), which is typical hallmark of the last stages of post‐infarction cardiac fibrosis, as highlighted in previous studies and in clinical reports.^[^ ^18^ ^]^ The role of both TGF‐β1 and mechanical stretching in triggering ECM production was also confirmed by the analysis of deposited Collagen Type I, Fibronectin and Aggrecan proteins (Figure 3). Respect to static conditions, where proteins appeared mostly at intracellular level, in stimulated microtissues, especially under the application of cyclic mechanical strains, ECM matrix became more homogenous and denser throughout the whole microtissues. These results were in agreement with data previously obtained by our research group,^[^ ^14^, ^15^ ^]^ demonstrating that a cyclic uniaxial strain of 10–12% at 1 Hz to neonatal rat CFs and/or CMs embedded in fibrin gel was able to trigger in vitro cardiac fibrosis hallmarks (e.g., cell proliferation, expression of pro‐fibrotic genes and abnormal ECM deposition). This is also in line with the work by Kong at al.,^[^ ^42^ ^]^ who exploited a 1 Hz cyclic compression with 5–20% gradient intensities, to induce phenotypic transition to neonatal rat CFs embedded in gelatine Metacrylate (GelMA) hydrogels.

Fibroblasts transition into myofibroblast phenotype, together with enhanced ECM protein deposition, may be induced by the activation of several signaling pathways.^[^ ^43^ ^]^ Activation of RhoA/ROCK2 pathway following mechanical stimulation has been previously reported to cause the phenotype switch of fibroblasts into myofibroblasts.^[^ ^44^ ^]^ Moreover, RhoA and ROCK2 enhanced expression has been correlated to increased Fibronectin and Collagen I deposition.^[^ ^45^ ^]^ In our uScar model, we observed increased RhoA/ROCK2 expression (Figure 3c.i,ii) following mechanical stimulation and TGF‐β1 administration, and this correlates with myofibroblast phenotype acquisition and higher ECM deposition. Moreover, we investigated the Hippo pathway, which activation is known to be regulated by mechanical strains and cell‐ECM interactions.^[^ ^46^ ^]^ Recent studies have identified YAP/TAZ as one of the molecular mechanisms underlying mechanotransduction. YAP and TAZ are two transcriptional co‐activator proteins, known as downstream effectors of the Hippo pathway, that play key roles in many biological processes through the activation of TEAD transcription factor.^[^ ^46^ ^]^ In our study, we observed increased YAP, TAZ and TEAD expression (Figure 3c,iii–v) in uScar models following mechanical stimulation and TGF‐β1 administration, which may be correlated to the activation of RhoA/ROCK2 signaling pathway and in turn to the observed increased cell interaction and ECM protein deposition. The application of cyclic strain, whether alone or in combination with TGF‐β1, also led to an increased expression of GSK‐3β, which is known for its pivotal role in the WNT/β‐catenin signaling pathway.^[^ ^38^ ^]^ Specifically, in the absence of Wnt, GSK‐3 β participates in the degradation complex responsible for β‐catenin degradation, but when Wnt ligands bind to frizzled receptors, the complex disassembles, allowing β‐catenin to translocate into the nucleus and activate specific downstream genes (e.g., COL1, PAI‐1).^[^ ^47^ ^]^ Although our model displayed increased expression of *COL1A2* in dynamic microtissues compared to static controls, and elevated expression of *COL1A2* and *PAI‐1* in microtissues subjected to combined mechanical stimulation and TGF‐β1, further investigations (e.g., phosphorylation status of β‐catenin) are necessary to conclusively establish the involvement of the WNT/β‐catenin pathway in our uScar model. The increased expression of GSK‐3β, may indicate an interaction between the WNT/β‐catenin pathway and the canonical TGF‐β pathway.^[^ ^47^ ^]^ Recent evidence^[^ ^48^ ^]^ suggests indeed that GSK‐3β can inhibit the TGF‐β pathway by directly interacting with SMAD‐3, thereby exerting a negative regulatory influence on the protein stability and enzymatic activity. This may suggest that in our dynamic model, TGF‐β1 mainly acts through the non‐canonical pathway, specifically mediated by the previously mentioned RhoA/ROCK2 signaling, further confirming their involvement in induction of fibrotic traits in uScar. Unaltered expression levels of TGF‐β receptors, specifically TGFβ‐R1 and TGFβ‐R2, in the dynamic and dynamic + TGFβ1 conditions, when compared to the control group, is in agreement with findings by Watson et al.^[^ ^39^ ^]^ Specifically, they subjected 2D human cardiac fibroblasts to biaxial mechanical stimulation in the presence or absence of 10 ng mL^−1^ TGFβ1. In their study, cyclical mechanical stretching mitigated the effectiveness of TGFβ1 in inducing myofibroblast differentiation, marked by increased BNP and NPRA expression in human cardiac fibroblasts. Conversely, in our 3D model, uniaxial mechanical strain appeared to enhance the efficacy of TGFβ1 in driving myofibroblast differentiation and interestingly, the expression levels of BNP were reduced in both dynamic and dynamic + TGFβ1 conditions, while NPRA expression decreased when both stimulations were combined. This contrasting trend may be attributed to differences in the environment (2D versus 3D and fibronectin‐coated membrane versus 3D fibrin gel), and the type of mechanical stimulation applied (2D biaxial for 72 h versus 3D uniaxial for 7 days). Nevertheless, the potential involvement of BNP and NPRA in modulating myocardial fibrosis was reaffirmed.

By performing the experiment with human cells, our results corroborated the suitability of uScar to generate a representative human cardiac fibrotic model without the use of exogenous cytokines, which may create a bias affecting the screening results of potential anti‐fibrotic drug candidate acting on the same pathway. The capability of uScar model to predict anti‐fibrotic effect of small molecules was further proven in the conducted drug screening tests. Pirfenidone, a commercially available drug for treating idiopathic pulmonary fibrosis,^[^ ^49^ ^]^ was confirmed to act as an anti‐fibrotic compound also in our model, highlighting the possibility to exploit our human set‐up for drug repositioning. Specifically, Pirfenidone administration at the beginning of the culture led to a reduction in the number of α‐SMA positive cells compared to the purely dynamic controls, reaching values closer to static microtissues, and to a suppression of ECM production, especially for Collagen type‐I (Figure 4b,c). These data partially supported the work of Mastikhina and colleagues,^[^ ^18^ ^]^ that previously reported efficacy of Pirfenidone in reducing cardiac fibrosis hallmarks in a 3D in vitro human model. The authors showed a drug‐related decrease in tissue stiffness, B‐type natriuretic peptide secretion, and fibrosis‐related microRNA secretion, but not a Collagen type‐I content reduction. This may be related to the different set‐up exploited by the authors, which encompassed both FBs and CMs or to the exploitation of TGFβ1 as stimulus to trigger the fibrotic phenotype in the model. TGFβ1 indeed may contrast with the drug mechanism of action as previously reported in the study of Bracco Gartner et al.,^[^ ^50^ ^]^ where the authors demonstrated that the drug has anti‐fibrotic effects in a mechanically induced 3D human model of cardiac fibrosis, but it is not able to revert all the TGF‐β1 biochemically induced changes. This confirms the importance of providing alternative methods (i.e., mechanical cues) to the exogenous cytokine addition for developing models aimed at elucidating the potential anti‐fibrotic effect of compounds. In the same context of use, Tranilast, an antihistamine suppressing the expression and activity of TGFβ that was initially developed to treat bronchial asthma and that was found effective in cardiomyopathy animal models,^[^ ^51^ ^]^ significantly reduced both myofibroblasts transition and ECM deposition (Figure 4d), as observed through immunofluorescence staining of α‐SMA, Collagen type I, Fibronectin and Aggrecan. These results confirmed that mechanical stimulation alone could enhance fibrotic remodeling of the tissue, which was instead attenuated by the drug administration, in accordance to previously reported studies in vitro and in vivo.^[^ ^42^, ^52^ ^]^

The versatility of uScar model and the importance to apply our approach in different context of uses was further confirmed by testing an advanced direct reprogramming therapy, previously employed and optimized by Paoletti et al. to reprogram 2D and 3D cultured AHCFs into iCMs.^[^ ^30^, ^32^ ^]^ Specifically, a combination of four miRNAs (miRcombo) were loaded into a new DE‐DOPE lipoplexes developed by Nicoletti and Paoletti et al.^[^ ^33^ ^]^ The DE‐DOPE lipoplexes indeed was demonstrated to possess a higher miRNA delivery ability and higher cytocompatibility compared to the traditional DharmaFECT1 agent, enhancing in vitro direct reprogramming of AHCFs into iCMs.^[^ ^26^, ^28^, ^29^ ^]^ In this work DE‐DOPE/miRcombo lipoplexes were thus exploited to trigger direct cell reprogramming, by transfecting AHCFs before their embedment into 3D models, or by delivering miRcombo to pre‐formed 3D uScar fibrotic microtissue models. A 24 h transfection time was applied, coherently with previous studies,^[^ ^30^, ^32^, ^33^ ^]^ while miRNA concentration was scaled‐up considering the higher cell number present in the 3D models. To assess the miRcombo therapy as a fibrotic preventing strategy in an in vivo resembling environment (i.e., 3D and mechanically active), 2D miRcombo‐transfected AHCFs were embedded in a 3D fibrin hydrogel in the uBeat Stretch Platform and, after 1 week of static culture, were subjected to dynamic (i.e., under cyclic mechanical stimulation) or static culture conditions. 2D transfected cells incorporated in the fibrin gel and cultured in 3D for 15 days in static conditions showed enhanced levels of cardiomyocyte markers, such as TNNT2 and MYL7 gene expression and α‐Sarcomeric Actinin production (Figure 5b,d). These results confirmed previously reported in vitro findings employing murine or human CFs in 2D^[^ ^32^, ^33^, ^53^ ^]^ and neonatal mouse^[^ ^27^ ^]^ or human^[^ ^30^ ^]^ CFs in fibrin‐based 3D hydrogels. In contrast, for the first time, the uniaxial cyclic strain provided in 3D was demonstrated responsible to prevent the CM‐like phenotype switch of 2D transfected AHCFs and to instead promote the formation of fibrotic microtissues with enhanced level of *COL1A2* gene expression and Collagen type‐I production (Figure 5c,e). Mechanical stimulation was here provided at physiological level to mimic the in vivo‐like mechanical stress that an envisioned injectable miRNA therapy would encounter once administered to the heart of the patient. Our results highlighted the fundamental importance of reproducing this mechanical cue in vitro, to more accurately evaluate the efficacy of therapies under development. The data agree with results by Sia et al.,^[^ ^31^ ^]^ reporting that cyclic mechanical stimulation (10% strain, 1‐Hz frequency) for 10 days in 2D condition reduced the percentage of reprogrammed mouse fibroblasts, compared to static control conditions. Moreover, miRcombo was found to be only slightly effective in inducing direct cell reprogramming when administered to 3D cardiac tissue model developed in static conditions, as evidenced by α‐Sarcomeric Actinin quantification (Figure 6b,c). In our experiments CFs could spontaneously express cardiomyocyte‐related genes, such as *TNNT2* after 15 days in static 3D culture condition, as previously demonstrated by Paoletti et al.^[^ ^30^ ^]^ *TNNT2* and MYL7 were also detected 21 days post transfection with negmiR in static condition. All these results are also in agreement with findings by Dr. Victor Dzau, the pioneer scientist of reprogramming miRcombo. Indeed, cardiac and tail‐tip mouse fibroblasts were induced to express cardiac genes in vitro, and 3D culture by itself was sufficient to augment the expression of cTnT and α‐Sarcomeric Actinin.^[^ ^27^ ^]^ Similar findings have been reported in other studies,^[^ ^54^ ^]^ including those by Paoletti et al.,^[^ ^30^ ^]^ further corroborating the notion that 3D in vitro environment alone can influence the plasticity of somatic cells.

Of note, miRcombo was revealed effective in mitigating the fibrotic traits acquired in uScar microtissues developed under dynamic culture condition (Figure 6b,d). These results align with in vivo studies, which have previously demonstrated that miRcombo administration, using viral vectors or nanoparticles, in mice subjected to myocardial infarction was able to strongly reduce Collagen I deposition, decreasing scar area and improving heart functions, despite the reported low reprogramming efficiency in vivo (≈1.5%).^[^ ^55^ ^]^ Indeed, how miRcombo works in inducing cell reprogramming and scar size reduction is still an open question. Wei et al.^[^ ^13^ ^]^ have reported that miR‐133 delivery was able to decrease α‐SMA expression and Collagen I deposition in lung fibroblasts, thus reducing cell transition into myofibroblast and pulmonary fibrosis alone or following TGF‐β treatment. Our finding that miRcombo more effectively reduced fibrotic characteristics in mechanically stimulated microtissues further supports previous in vivo observations, where the transfection efficiency was revealed higher in activated CFs compared to quiescent ones.^[^ ^56^ ^]^ Despite promising, miRcombo was not able to effectively induce a CM‐like phenotype switch in the cells. This remarkably corroborates a few open questions in the field, demanding for more reliable and predictive in vitro human models as useful tools to evaluate the best clinical application window for efficient direct reprogramming of fibroblasts into iCMs, to achieve myocardial regeneration.^[^ ^57^ ^]^ Immunofluorescence analysis did not show the production of cTnT in the model at 7 days post‐transfection, probably due to the short culture time. Indeed, previous reports by Paoletti et al.^[^ ^30^, ^32^ ^]^ and Li et al.^[^ ^27^ ^]^ showed cTnT production at 14 days post‐transfection with miRcombo. Therefore, despite the mechanical stimulation could represent a valid tool for in vitro maturation of already generated CMs, as previously found for CMs differentiated from induced pluripotent stem cells (iPSCs), its application to drive such maturation in reprogrammed cells will be probably possible only once direct reprogramming efficiency has been optimized.^[^ ^58^ ^]^

Overall, results from this work suggested that the application of an in vivo like mechanical stimulation under 3D culture conditions is fundamental to assess the efficacy of drugs and new advanced therapies, such as direct reprogramming.

## 4 Conclusions

In this study, a 3D human in vitro model of cardiac fibrosis, uScar, was generated by taking advantage of a microfluidic platform combining biochemical and biomechanical stimuli. The cyclic physiological uniaxial strain provided to 3D AHCFs microtissues was sufficient to trigger in the model some of the main hallmarks of cardiac fibrosis, from fibroblast phenotype switch to ECM deposition. The model was demonstrated able to be applied for efficacy tests on commercially available drugs and advanced miRNA therapies aimed at the direct reprogramming of fibroblasts into cardiomyocytes. The work highlights the importance of incorporating different in vivo like stimulations (i.e., mechanical cues) into 3D in vitro models, as they may potentially interfere with the efficacy of a therapy once administered to the patients.

## 5 Experimental Section

### Platform Layout and Fabrication

The beating‐heart‐on‐chip platform was composed by three superimposed layers: the actuation layer on top, the chamber layer in the center and a coverslip at the bottom. The actuation layer encompassed three actuation chambers connected through a microchannel, whose termination was coupled to a pneumatic actuation system. The chamber layer contains three independent cell culture chambers, each composed by a central channel (300 and 150 µm high) for the gel confinement and two side channels for the cell medium supply, divided by two rows of hanging shield‐shaped pillars. The pillars allowed the cell‐laden gel confinement in the cell channel and the medium diffusion through the gaps between them. Cell‐laden hydrogel was injected into the central channel through a 1 mm inlet, while the medium was delivered into the lateral channel from four 5 mm reservoirs, located at the extremities. Thanks to a 50 µm gap below the pillars, the cell culture chambers can be moved toward the coverslip, producing a uniaxial strain of 10% in the microtissue.^[^ ^35^ ^]^ The three actuations were pressurized at once, enabling the stretching of three independent microtissues simultaneously. The actuation and the cell culture layer were made of polydimethylsiloxane (PDMS, Sylgard 184, Dow Corning, 1:10 w w^−1^ of cross‐linker pre‐polymer proportion). The three layers were aligned and linked through air plasma bonding (plasma cleaner Harrick Plasma, PDC‐002). Before using, the devices were autoclaved at 134 °C to be sterilized and let dry for at least 24 h.

### AHCFs Expansion and uScar Cardiac Fibrotic Microtissues Formation

Normal human atrial cardiac fibroblasts (AHCFs) were purchased at P2 from Lonza (CC‐2903; batch: 000 0662121; male donor, 48 years old), expanded using Fibroblasts Growth Medium‐3 (Lonza, CC‐4526) containing 10% fetal bovine serum (FBS), 1% insulin, 1% human basal fibroblast growth factor (hFGF‐B) and 1% gentamicin and freezed at P4. For the experiments, AHCFs were thawed, seeded at 20.000 cells cm^−2^ on 2D plates precoated with a 10 µg mL^−1^ porcine gelatin solution (Sigma‐Aldrich), and expanded for 5 days by using complete medium made of DMEM high glucose (GIBCO) supplemented with 10% FBS, 1% HEPES (GIBCO) and 1% Pen‐Strep‐Glutamine (GIBCO). To generate the microtissue within the heart‐on‐chip, AHCFs were embedded in fibrin hydrogel (i.e., 15 × 10^6^ cell mL^−1^) and injected into the cell culture chambers. Briefly, two separated solutions were prepared and kept on ice: a fibrinogen solution containing 20 mg mL^−1^ of fibrinogen (Sigma Aldrich) dissolved in Phosphate Buffered Saline (PBS, GIBCO), and a mix solution containing 5 U mL^−1^ of thrombin (Baxter) in DMEM high‐glucose where AHCFs were resuspended at 30 × 10^6^ cells mL^−1^. The two solutions were mixed in a 1:1 ratio, inoculated into the platforms and let to cross‐link for 8 min in the incubator. The obtained 3D microtissues were hydrated with complete medium supplemented with 2 mg mL^−1^ of 6‐aminocaproic acid (ACA) and cultured for 7 days in the four different conditions: static, dynamic (i.e., cyclic mechanical stimulation at 1 Hz, providing a 10% uniaxial strain), static + TGFβ1 (i.e., 5 ng mL^−1^) and dynamic + TGFβ1 (i.e., cyclic mechanical stimulation at 1 Hz, providing a 10% uniaxial strain with 5 ng mL^−1^ of TGFβ1). Cell culture medium was changed the day after seeding and every other day. ACA content was diminished as it follows: day 1 at 2 mg mL^−1^, day 3 at 1.4 mg mL^−1^, day 5 and day 7 at 1 mg mL^−1^. The fibrotic traits onset was assessed by studying the expression of pro‐fibrotic genes through real time quantitative polymerase chain reaction (RT‐qPCR) and the production of specific fibrotic proteins by means of immunofluorescence staining.

### Live/Dead Staining and Cell Viability Quantification

After 7 days of culture in static and dynamic (i.e., cyclic uniaxial strain at 10%) conditions, a Live/Dead assay (Invitrogen, TermoFisher Scientific, USA) was performed to evaluate AHCFs cell viability in the beating heart‐on‐chip. The assay was performed following the manufacturer's instructions. Briefly, the cell culture medium was removed from the wells and the microtissues were washed twice with warm PBS and incubated with the live/dead solution (i.e., 2 µm Calcein and 4 µm ethidium homodimer) for 15 min at 37 °C and 5% CO_2_ in the dark. Images were acquired by means of an inverter microscope (Motic, USA) using 10× and 20× magnification objectives. Live cells were visualized using FITC filter (green), while dead cells nuclei using Texas Red filter (red). The images were analyzed using ImageJ (National Institutes of Health). Briefly, three regions of interest (ROI) in three independent microtissues of each condition were selected and their live and dead images were merged. The green stained (i.e., live) and red stained (i.e., dead) cells were manually counted and the percentage in cell viability or mortality was calculated. The data were reported as means ± standard deviation of n = 3 samples.

### Drug Screening Tests in uScar Cardiac Fibrotic Microtissues

For the drug screening tests, fibrotic microtissues were obtained by providing a cyclic uniaxial strain of 10% at 1 Hz to AHCFs embedded in fibrin (i.e.,10 mg mL^−1^ of fibrinogen and 1.5 u mL^−1^ of thrombin) for 7 days, as previously described. Pirfenidone (1 mM) or Tranilast (50 µm) were administered from the beginning of the culture, so to assess their efficacy in preventing mechanically‐driven traits of cardiac fibrosis. Static and dynamic conditions without drugs were used as controls. Medium changes was performed in all conditions the day after seeding and every other day by using complete medium supplemented with 2 mg mL^−1^ of ACA. The compounds were re‐administered at each medium change at the initial concentration, while ACA content was diminished as it follows: day 1 at 2 mg mL^−1^, day 3 at 1.4 mg mL^−1^, day 5 and day 7 at 1 mg mL^−1^. Immunofluorescence staining of specific fibrosis‐related proteins was used to evaluate the microtissues’ fibrotic phenotype switch and the level of matrix deposition.

### 2D AHCFs Reprogramming and 3D Culture in the Beating Heart‐on‐Chip

To assess fibroblast reprogramming efficacy after mechanical stimulation in the uScar platform, a previously developed combination of miRNAs,^[^ ^33^ ^]^ that was found effective in 2D and in 3D to directly reprogram fibroblast into induced cardiomyocyets (iCMs)^[^ ^30^, ^32^ ^]^ was used to transfect AHCFs. In details, 110.000 AHCFs were plated in 6 multiwell plates in DMEM High Glucose with 10% FBS and 1% glutamine (Sigma‐Aldrich) 24 h before transfection. A mix of four different miRNA mimics (mirVana miRNA mimic, Life Technologies) termed miRcombo (i.e., miR‐1, miR‐133, miR‐208, and miR‐499) was encapsulated in DE‐DOPE liposomes ([2‐(2,3‐didodecyloxypropyl)‐hydroxyethyl] ammonium bromide, DE^[^ ^31^ ^]^ and L‐alpha‐dioleoylphosphatidylethanolamine, DOPE, Sigma‐Aldrich), to form DE‐DOPE/miRcombo lipoplexes as previously reported.^[^ ^33^ ^]^ Briefly, empty DE‐DOPE liposomes were prepared using the thin lipid film‐hydration method.^[^ ^59^ ^]^ The DE‐DOPE lipid film was hydrated with Milli‐Q water to a final concentration of 1 mg mL^−1^ of lipids. Then, DOPE/miRcombo lipoplexes were prepared via spontaneous electrostatic interaction at amino to phosphate groups (N/P) ratio of 3 (miRcombo, 0.7 µg and DE‐DOPE, 6 µg) as previously optimized. DE‐DOPE/miRcombo lipoplexes were administered to the 2D cultured AHCFs at a final concentration of 25 nmol L^−1^ in DMEM High Glucose supplemented with 10% FBS and 1% glutamine. NegmiR (mirVana miRNA Mimic, Negative Control #1, Life Technologies) incapsulated in DE‐DOPE lipoplexes at the same concentration was used as negative control. After 24 h, the transfectant solution was removed and the cells were harvested to be inoculated into the beating heart‐on‐chip. Briefly, transfected cells were embedded in fibrin hydrogel (i.e., final concentration of 10 mg mL^−1^ fibrinogen and 2.5 U mL^−1^ of thrombin) at a 15 × 10^6^ cells mL^−1^ density and cultured within the platform in static conditions for 7 days. After this period, a mechanical stimulation was started only on miRcombo‐transfected cells. Static samples from miRcombo and negmiR transfected AHCFs were used as controls. DdPCR and immunofluorescence staining were used to assess whether the transfected cells were able to maintain a cardiomyocyte‐like phenotype or their switch into the fibrotic counterpart.

### Direct Reprogramming of uScar 3D Cardiac Fibrotic Microtissues

To investigate the capability of the gene therapy to revert the fibrotic traits, the uScar fibrotic microtissues were obtained as previously described by providing to the 3D AHCFs laden fibrin gels (i.e., 15 × 10^6^ cells mL^−1^ embedded in 10 mg mL^−1^ fibrinogen and 2.5 U mL^−1^ thrombin) a cyclic mechanical strain at 10% for 7 days, while static microtissues were used as controls. Medium changes were performed the day after seeding and every other day by using complete medium supplemented with 2 mg mL^−1^ of ACA. ACA content was diminished as follows: day 1 at 2 mg mL^−1^, day 3 at 1.4 mg mL^−1^, day 5 at 1 mg mL^−1^. On day 6, the cell culture medium was changed with DMEM High Glucose supplemented with 10% FBS and 1% glutamine. At day 7, miRcombo (i.e., miR‐1, miR‐133, miR‐208, and miR‐499) or negmiR (i.e., vehicle negative control) were encapsulated in DE‐DOPE lipoplexes and administered to the microtissues at 25 nmol L^−1^ in DMEM High Glucose supplemented with 10% FBS and 1% glutamine. After 24 h, the transfectant solutions were removed and the devices were cultured for additional 15 days in static conditions. DdPCR analyses and immunofluorescence staining were used to assess the expression of iCMs‐related genes and iCMs‐ or FBs‐related proteins levels after 7 or 15 days of transfection.

### RNA Extraction and Quantitative Real Time

At the end of the culture, microtissues were washed twice in PBS, harvested from the beating heart‐on‐chip and collected in tryzol (Sigma Aldrich) to proceed with the RNA extraction, as previously described.^[^ ^34^ ^]^ RNA concentration and quality were quantified using Nanodrop spectrophotometer (TermoFisher, USA). Reverse transcription of RNA into cDNA was performed using a Superscript III Reverse Transcriptase Kit (TermoFisher, USA) following the manufacturer's protocol. Quantitative Real Time Polymerase Chain Reaction (qRT‐PCR) through Fast Real‐Time PCR System (Applied Biosystems, USA), was used to assess the effect of different stimulation (i.e., static, dynamic, static + TGFβ1, dynamic + TGFβ1) provided within the beating heart‐on‐chip in triggering cardiac fibrotic traits after 7 days of culture. Specifically, the expression of Collagen type I (*COL1A1*, ID assay: Hs00164004_m1) and Collagen type III (*COL3A1*, ID assay: Hs00943809_m1) genes, indicators of ECM production, and of TGF‐β1 (*TGFB1*, ID assay: Hs00998133_m1) gene, a key marker of fibrosis was evaluated. The expression levels of each gene were normalized to glyceraldehyde‐3‐phosphate dehydrogenase (*GAPDH*, ID assay: Hs02758991_g1) housekeeping gene and calculated using the 2‐ΔCt method. Data were represented ad mean ± standard deviation of at least n = 3 independent samples.

### Droplet Digital‐ Polymerase Chain Reaction

Reverse transcription of RNA into cDNA was performed using a High‐Quality cDNA Reverse Transcription kit (Applied Biosystems, USA), following the manufacturer's protocol. DdPCR was performed to assess the expression of TNNT2 (ID assay: dHsaCPE5052344), MYL7 (ID assay: qHsaCEP0050426), COL1A2 (ID assay: dHsaC‐PE5031597), TGFBR1 (ID assay: dHsaCPE5048610), TGFBR2 (ID assay: dHsaCPE5054214), NPPB (ID assay: dHsaCPE5035504), NPR1 (ID assay: dHsaCPE5040421), RHOA (ID assay: dHsaCPE5032725), ROCK2 (ID assay: dHsaCPE5056316), MKL1 (ID assay: dHsaCPE5048472), SRF (ID assay: dHsaCPE5052590), YAP1 (ID assay: dHsaCPE5030690), WWTR1, TAZ (ID assay: dHsaCPE5191727), TEAD1 (ID assay: dHsaCPE5042644), CTGF (ID assay: dHsaCPE5025868), SERPINE1, PAI‐1 (ID assay: dHsaCPE5034214), CTNNB1 (ID assay: dHsaCPE5040214), GSK3β (ID assay: dHsaCPE5045593) using ddPCR Supermix for probes without dUTP. Droplet generation was performed according to manufacturer's instructions. Thermal‐cycling conditions were 95 °C for 10 min (1 cycle), 94 °C for 30 s and 55 °C for 30 s (40 cycles), 98 °C for 10 min (1 cycle), and a 4 °C infinite hold. DdPCR protocol continued as described above. GAPDH (ID assay: dHsaC‐PE5031597) was used as a housekeeping gene to perform quantitative normalization. Results were reported as the concentration (cDNA copies µL^−1^) of the gene of interest respect to the mean concentration (cDNA copies µL^−1^) of GAPDH. No template control with water was included in each assay. Data are presented as mean ± SEM. Experiments were performed in triplicate and repeated two times.

### Immunofluorescence Staining and Quantification of Phenotype Switch and Matrix Deposition

Immunofluorescence staining was performed within the platforms at the end of the culture and was exploited to evaluate the fibroblast to myofibroblast transition (i.e., α‐Smooth Muscle Actin), the matrix deposition (i.e., collagen I, fibronectin, and aggrecan) and the CM (i.e., Cardiac troponin T and α‐Sarcomeric‐Actinin) and FB (DDR2) phenotypes. Briefly, samples were incubated for 30 min in 4% paraformaldehyde and later permeabilized for 1 h at room temperature with 0.1% v v^−1^ Triton X‐100 (Thermo Fisher Scientific) in a solution of 2% w v^−1^ bovine serum albumin (BSA, Sigma Aldrich) dissolved in PBS, to block nonspecific binding. Primary antibodies were prepared in a solution of 0.5% w v^−1^ BSA. Cardiac microtissues were incubated overnight at 4 °C in defined primary antibody solutions, prepared accordingly to manufacturer. Specifically, anti‐α‐SMA mouse monoclonal IgG2a antibody (Termofisher) was diluted 1:500, anti‐collagen type I mouse monoclonal IgG1λ antibody (Santa Cruz Biotechnologies) was diluted 1:100, anti‐aggrecan mouse monoclonal IgG1 antibody (Santa Cruz Biotechnologies) was diluted 1:200, anti‐fibronectin mouse monoclonal IgG1k antibody (Santa Cruz Biotechnologies) was diluted 1:200, anti‐troponinT rabbit monoclonal IgG antibody (Termofisher) was diluted 1:200, anti‐DDR2 mouse monoclonal IgG2a antibody (Termofisher) was diluted 1:200 and anti‐α‐sarcomeric‐actinin (Abcam) was diluted 1:200. After incubation, the microtissues were washed twice in 0.5% w v^−1^ BSA and then incubated with secondary antibody solutions for 6 h at 4 °C in the dark. The solutions were made by resuspending 546 Goat Anti‐mouse IgG1 (Alexa Fluor, 1:200 dilution), 488 Goat Anti‐rabbit IgG (H+L, Alexa Fluor, 1:250 dilution) and 555 Goat Anti‐rabbit IgG (H+L, Termofisher, 1:250 dilution) in 0.5% w v‐1 BSA. 4′,6‐diamidino‐2‐phenylindole (DAPI, Invitrogen, 300 nm) nuclear counterstaining was used to identify cell nuclei. Nikon Eclipse Ti2 spinning disk microscope and NIS‐Elements software (Nikon) were used to image the samples at 20× and 40× magnification.

To quantify the phenotype switch of the cells, each z‐stack was projected onto a single z‐plane and the background of the obtained image was cleaned. DAPI image was merged with the corresponding α‐SMA image (i.e., blue color for DAPI, green color for α‐SMA) and a specific ROI was chosen, so to manually count the number of α‐SMA positive cells and the total cell nuclei to assess the percentage of α‐SMA positive cells over the total number of cells. Similarly, to quantify the ECM deposition, each z‐stack was projected onto a single z‐plane, the background of the obtained image was cleaned and ROIs were selected. DAPI images were used to manually count the total cell number, while Collagen I, Aggrecan or Fibronectin images were analyzed so to measure the fraction of the positive stained area. The percentage of matrix production was then calculated by normalizing the percentage of stained area to the total cell number. Data were represented ad mean ± standard deviation.

## Conflict of Interest

Roberta Visone, Marco Rasponi and Paola Occhetta share equities in BiomimX Srl.

## Supporting information

Supporting Information

## Data Availability

The data that support the findings of this study are available from the corresponding author upon reasonable request.
