# Supplementary material for: In Vitro Mechanical Stimulation to Reproduce the Pathological Hallmarks of Human Cardiac Fibrosis on a Beating Chip and Predict The Efficacy of Drugs and Advanced Therapies
Source: Adv Healthc Mater. 2023 Nov 27;13(4):2301481. doi: 10.1002/adhm.202301481 (PMC11468947; doi:10.1002/adhm.202301481)
Supplement: Supplementary file 1 — Supporting Information [file ADHM-13-2301481-s001.pdf]

ADVANCED HEALTHCARE MATERIALS

Supporting Information

for *Adv. Healthcare Mater.*, DOI 10.1002/adhm.202301481

In Vitro Mechanical Stimulation to Reproduce the Pathological Hallmarks of Human Cardiac Fibrosis on a Beating Chip and Predict The Efficacy of Drugs and Advanced Therapies

Roberta Visone, Camilla Paoletti, Alessandro Cordiale, Letizia Nicoletti, Carla Divieto, Marco Rasponi, Valeria Chiono and Paola Occhetta*

Supplementary Informations

In vitro mechanical stimulation to reproduce the pathological hallmarks of human cardiac fibrosis on a beating chip and predict the efficacy of drugs and advanced therapies.

Roberta Visone[1,2*], Camilla Paoletti[3,4,*], Alesandro Cordiale[2], Letizia Nicoletti[3,4], Carla Divieto[5], Marco Rasponi[2,4,§], Valeria Chiono[3,4], Paola Occhetta[1,2]

1 BiomimX Srl, Milan, 20157, Italy

2 Department of Electronics, Informatics and Bioengineering, Politecnico di Milano, Milan, 20133, Italy

3 Department of Mechanical and Aerospace Engineering, Politecnico di Torino, Turin, 10129, Italy

4 Centro 3R (Interuniversity Center for the Promotion of 3Rs Principles in Teaching and Research), Italy

5 Istituto Nazionale di Ricerca Metrologica, Division of Advanced Materials and Life Sciences, Turin, 10135, Italy

*These authors equally contributed

§ Corresponding Author: marco.rasponi@polimi.it

Supplementary Figure 1

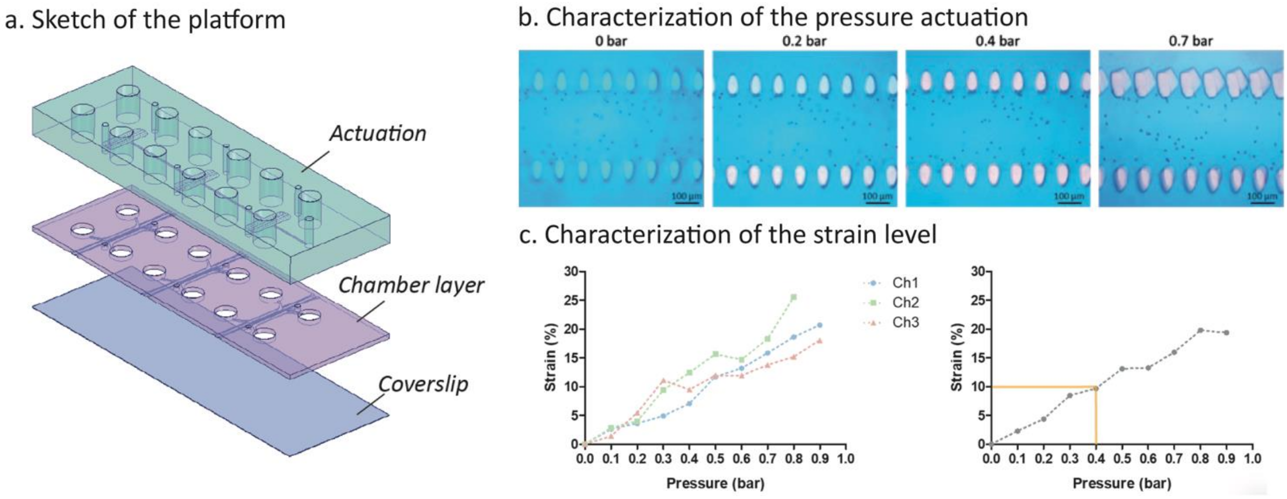

Supplementary Figure 1: Device representation and mechanical strain characterization. a) Representative sketch of the beating-heart-on-chip showing the assembly of the three different layers composing the platform. b) Characterization of the uBeat® mechanism[31]: the central part of the chamber was injected with fibrin laden polystyrene beads, the pressure in the actuation chamber was gradually increased (0-0.9 bar) and the movement of the pillars towards the coverslip was monitored. At 0.4 bar the pillars became completely white because in contact with the coverslip, while at 0.7 bar resulted deformed. c) Percentage of strain level in the microtissues within each chamber and their means, computed evaluating relative displacements of adjacent bead couples at the different actuation pressure. The physiological strain of 10% was achieved by proving a 0,4 bar actuation pressure.

Supplementary Figure 2

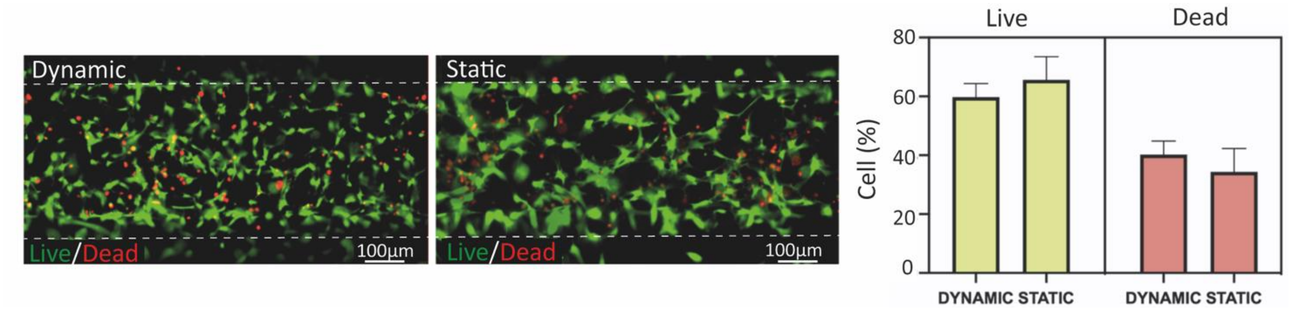

Supplementary Figure 2: Live/Dead staining in static and dynamic conditions and relative quantification of the percentage of viable and dead cells (means±standard deviation). Dotted lines represent the two parallel row of hanging pillars.

Supplementary Figure 3

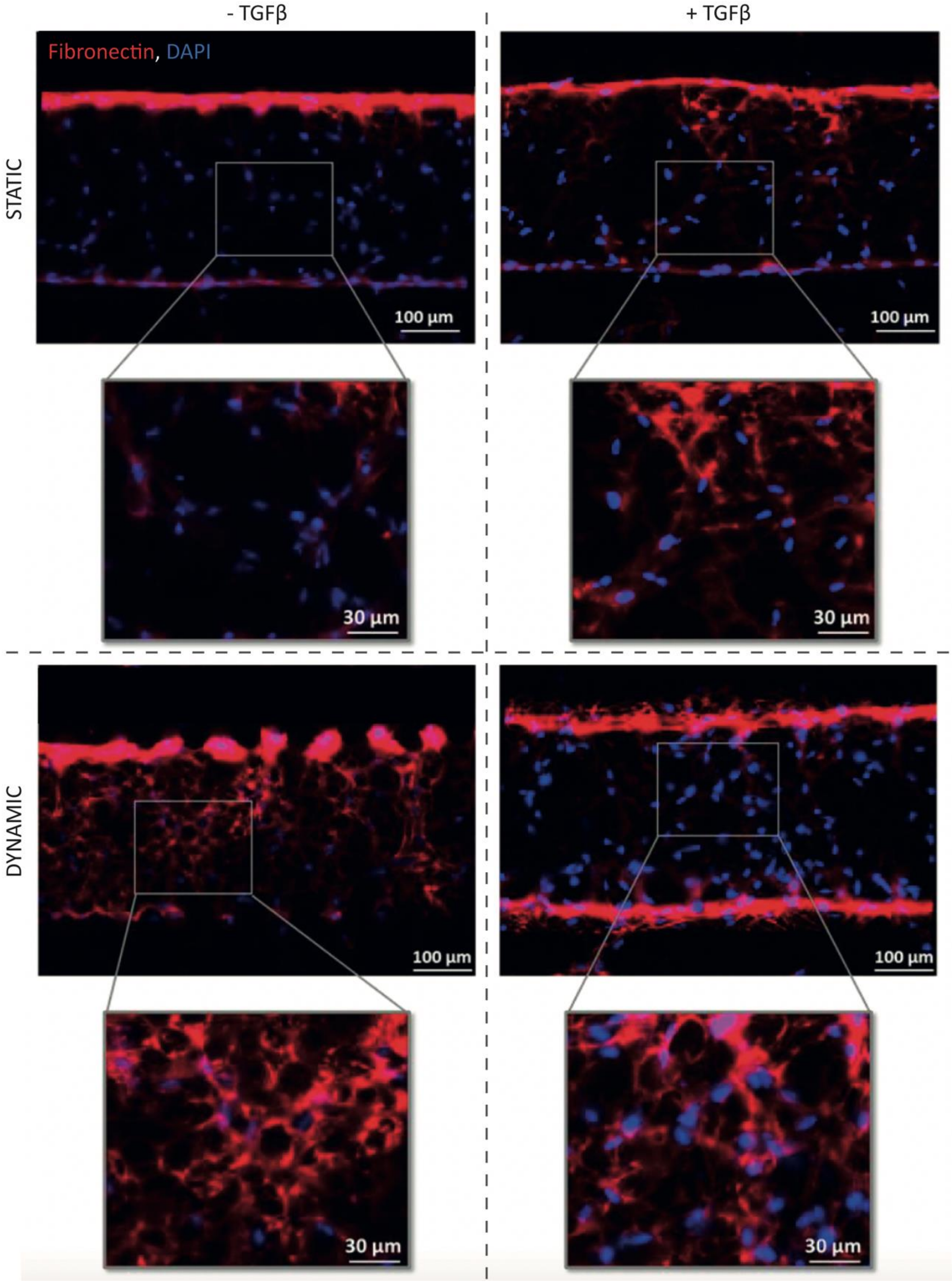

Supplementary Figure 3: Representative images of Fibronectin (red) immunofluorescent staining with DAPI counterstaining (blue), highlighting the presence of a massive protein deposition around the pillars in all the four tested conditions. Magnification of the central area of the channel allowed a proper visualization of the matrix deposition throughout the whole microtissue.

Supplementary Figure 4

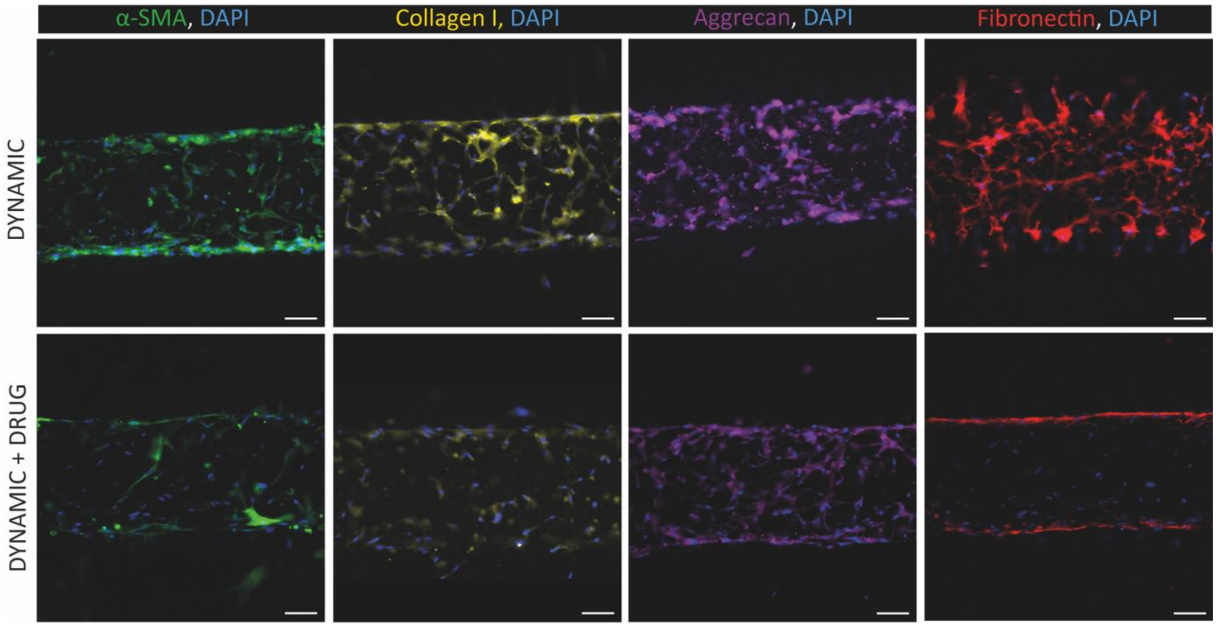

Supplementary Figure 4: Effect of Tranilast in preventing the fibrotic traits elicited by the mechanical stimulation in the uScar human model: Representative immunofluorescence images of activated fibroblasts (α-SMA-green) and ECM components, such as Collagen I (yellow), Aggrecan (purple) and Fibronectin (Red). Scalebar 100µm.

Supplementary Figure 5

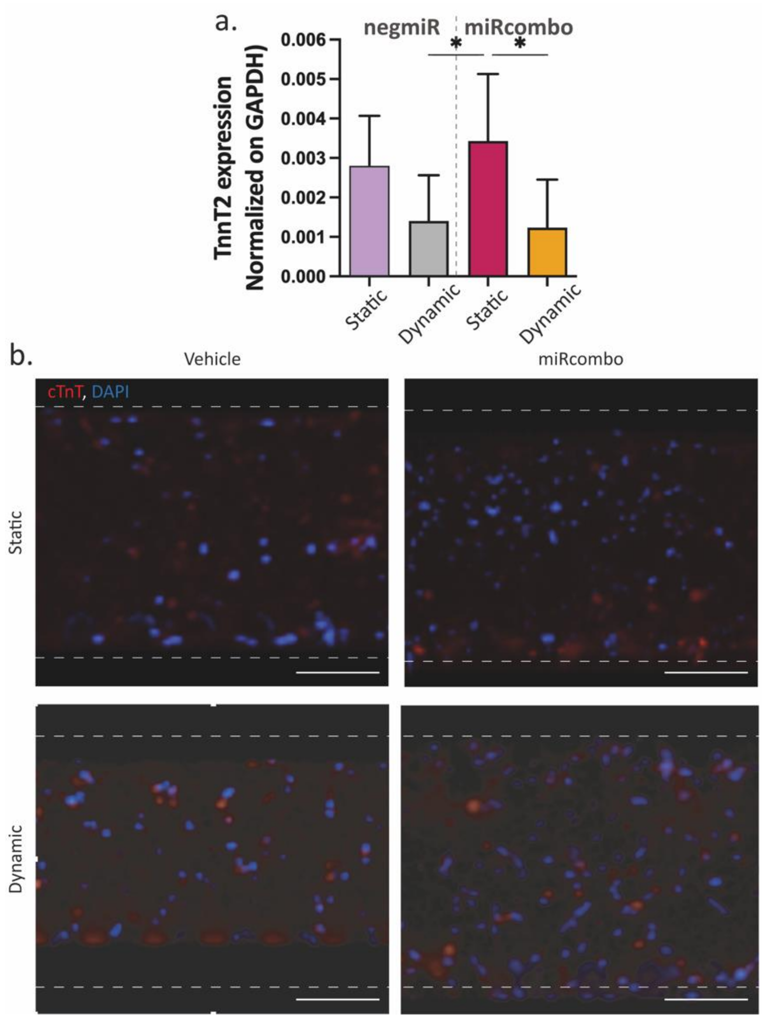

Supplementary Figure 5: a) mRNA level expression of cardiomyocyte typical markers (TNNT2) in AHCF microtissues cultured in static or dynamic conditions for 7 days, transfected with negmiR or miRcombo and then cultured for other 7 days in static conditions.;b) Representative images of TroponinT (red) immunofluorescent staining with DAPI counterstaining (blue), highlighting a mild presence of the protein in all the four tested conditions. Saclebar 100µm, dotted white line represent the position of the pillars.
